# The prevalence of abnormal semen parameters in male partners of women with anovulatory polycystic ovarian syndrome: a retrospective case–control study

**DOI:** 10.1007/s00404-024-07760-3

**Published:** 2024-10-04

**Authors:** Judith Aschauer, Johannes Ott, Clara Selzer, Stefan Ghobrial, Victoria Fitz, Marlene Hager

**Affiliations:** 1 Clinical Division of Gynecological Endocrinology and Reproductive Medicine, Department of Obstetrics and Gynecology, Medical University of Vienna, Spitalgasse 23, 1090 Vienna, Austria; 2 Division of Reproductive Endocrinology and Infertility, Department of OB/GYN, Massachusetts General Hospital, Harvard Medical School, Boston, MA USA

**Keywords:** Male factor, Ovarian stimulation, Polycystic ovary syndrome, Semen analysis, Infertility

## Abstract

**Purpose:**

Polycystic ovary syndrome (PCOS) is the leading cause of anovulatory infertility, often requiring ovarian stimulation in affected women attempting to conceive. Male partner semen quality and shared lifestyle factors can significantly impact reproductive outcomes. However, current international guidelines lack evidence-based recommendations on the necessity and timing of semen analysis for the fertility management of anovulatory PCOS women.

**Methods:**

In a retrospective case–control study, semen analysis results of male partners of 187 anovulatory PCOS women scheduled for ovarian stimulation were analyzed and compared to a control group of 76 male partners of women with bilateral tubal occlusion.

**Results:**

The prevalence of semen analysis results with at least one parameter classified as “borderline” and “pathological” among male partners of women with PCOS eligible to undergo ovarian stimulation was 51.3% and 22.5%, compared to 44.7% and 13.2% in the control group, respectively (*p* = 0.027). In the PCOS group, male body mass index (odds ratio, OR 1.478, *p* < 0.001), and smoking status (OR 6.228, *p* < 0.001) were significant predictors of pathological sperm test results, while no association was observed with any female characteristics (*p* > 0.05).

**Conclusion:**

The high frequency of pathological sperm analysis results provides lacking epidemiological data on semen quality in this population, emphasizing the critical need for early male fertility evaluation prior to ovarian stimulation in PCOS women. Moreover, our findings indicate that the risk prediction for abnormal semen quality cannot be based on the female’s data.

## What does this study add to the clinical work

**Table:** 

The findings of this study show how semen quality in male partners of PCOS women may also be reduced, aiming to offer more information for current PCOS guidelines to highlight the importance of early male fertility evaluation before ovarian stimulation, as a notably higher semen analysis abnormality rate was revealed in this patient cohort than in controls. Moreover, risk assessment of couples shared lifestyle factors on semen quality may guide anovulatory PCOS women on the utility of semen analysis before reproductive treatment.	

## Introduction

Polycystic ovarian syndrome (PCOS) is an endocrinologic and metabolic disorder in women of reproductive age with global prevalence estimates ranging from 6 to 10% [1]. The syndrome is commonly accompanied by irregularities in the menstrual cycle and recognized as the leading cause of anovulation, resulting in female infertility if left untreated [2]. Therefore, ovarian stimulation is necessary for reproductive management in many PCOS patients attempting to conceive. Modifiable lifestyle factors such as obesity and smoking can aggravate clinical symptoms and ovulatory dysfunction, consequently increasing the need for fertility interventions. The leading internationally accepted guideline for the diagnosis and treatment of polycystic ovarian syndrome recommends pharmacologic induction of ovulation in all women with an anovulatory PCOS in the absence of other evident and treatable infertility factors. Accordingly, the aromatase inhibitor letrozole and the selective estrogen receptor modulator clomiphene are recommended as the first- and second-line oral drugs for this indication [3].

Certainly, the likelihood of female conception is highly dependent on the fertility status of their male partner.

Sperm quality has also been associated with certain lifestyle habits and a man’s general health [4]. Male factors are estimated to play a role in limiting chances for pregnancy in almost half of all opposite-sex couples experiencing infertility, when infertility is defined as the inability of a couple to achieve pregnancy after a minimum of 12 months of regular, unprotected sexual intercourse, irrespective of the underlying etiologies that may be attributed to either partner [5, 6]. Initial evaluation typically involves quantitative and qualitative semen parameter analysis including sperm cell concentration, motility and morphology [7]. As recently reviewed, the majority of studies estimate that a male factor contributes to 30–50% of infertility cases [8]. Since infertility affects about 10–20% of all couples worldwide [9] and reproductive success is defined at the couple level, the etiology of infertility can be attributed to either or both members of the couple [10, 11]. In a large study, which included 8,500 infertile couples of 25 countries, female and male factor infertility was found in 37% and 8% of cases, respectively, whereas both partners contributed to the etiology of infertility in 35% [12]. Anovulation due to PCOS is a clear female cause of infertility. However, the question remains how often it would be accompanied by male infertility. Notably, there are differences in dietary habits between PCOS women and healthy female controls [13]. Cultural practices, which promote social connection and include family life, are often characterized by low-nutrient, high-calorie foods [14, 15]. Thus, male partners of PCOS women might also reveal unfavorable dietary patterns more frequently and, thus, could be affected by reduced semen quality more often, since male obesity is associated with a lower sperm quality [16]. Accordingly, we hypothesize that male partners of anovulatory PCOS women would reveal abnormal semen parameters more often than partners of women with another clear female cause of infertility, namely bilateral tubal blockage.

Notably, current international PCOS guidelines lack clear instructions on the appropriate timing and necessity of conducting semen analysis before commencing ovulation induction therapy. Moreover, the lack of research on semen quality patterns of male partners of women with anovulatory PCOS limits evidence-based recommendations regarding male fertility assessments during or prior to ovarian stimulation protocols in these patients. In an effort to address the gap in the literature, the main study objective was to analyze the rate of abnormal sperm test results among all male partners of women with PCOS who were considered eligible to undergo ovarian stimulation with letrozole or clomiphene. These data should be helpful when informing the couples of whether to conduct a semen analysis prior to initiating fertility treatment. Moreover, we aimed to search for both male or female predictive factors for abnormal semen analysis parameters in this population.

## Materials and methods

### Patient population

This retrospective study evaluated the prevalence of abnormal semen analysis results among 187 male partners of women aged 18–45 years with polycystic ovarian syndrome (PCOS) and ovarian stimulation after diagnostic evaluation for infertility at the Clinical Division of Gynecological Endocrinology and Reproductive Medicine, Department of Obstetrics & Gynecology of the Medical University of Vienna, Austria, from January 2016 to December 2022. Subjects were included in the analysis if their female partners were considered eligible for ovarian stimulation with letrozole or clomiphene and met the diagnostic criteria of PCOS according to the revised 2003 Rotterdam consensus statement [17]. Accordingly, PCOS was diagnosed in women who presented with at least two out of the following three features: oligo- or anovulation, signs of hyperandrogenism and/or polycystic ovaries on ultrasound. In detail and as reported previously [18], hyperandrogenism was defined as a testosterone level > 0.48 ng/mL, which is in accordance with the local normal ranges, and/or the presence of hirsutism; oligo-/anovulation was diagnosed based on the presence of oligo-/amenorrhea, i.e., a minimum cycle length ≥ 35 days in the last 3 months [19]; polycystic ovarian morphology (PCOM) was assessed using an Aloka Prosound 6 ultrasound machine (Wiener Neudorf, Austria; frequency range 3.0–7.5 MHz) and defined as a follicle number per ovary of ≥ 12 measuring 2–9 mm in diameter and/or ovarian volume of ≥ 10 ml, following international recommendations when using a transducer frequency less than 8 MHz [20, 21]. Women were excluded if other endocrine disorders potentially causing these symptoms were present. Semen analysis results were then compared to those of male partners of 76 women diagnosed with bilateral tubal occlusion by either hysterosalpingography (HSG), hysterosalpingo-contrast-sonography (HyCoSy) or laparoscopic chromopertubation, visiting the same clinic for fertility management.

The study was approved by the ethics committee of the Medical University of Vienna in January 2020 (IRB number 1602/2020).

### Parameters analyzed

Patient data were obtained using the AKIM-software (version 7, SAP Software Solutions Austria, Vienna; SAP-based patient management system at the Medical University of Vienna). The main outcome was the semen analysis result of the male partner.

Sperm samples were obtained through masturbation and assessed following laboratory protocols outlined in either the 2010 or 2021 editions of the World Health Organization (WHO) manual for human semen analysis [22, 23]. Importantly, these laboratory procedures described for assessing basic the semen parameters of sperm count, morphology and motility have undergone only minimal adjustments in the updated manual compared to its predecessor [24], making absolute values of the results comparable for further interpretation. All analyses were conducted in certified laboratories undergoing regular quality controls. Results were classified as abnormal if at least one sperm cell parameter fell below the lower reference limits identified by the 5th percentile of a fertile male population provided by the 2021 WHO guideline. “Decision limits” were used in accordance to the “Editorial Commentary on Draft of World Health Organization Sixth Edition Laboratory Manual for the Examination and Processing of Human Semen” [25] and several recent publications [26–30]. Details are provided in Table 1. For the final analysis, a semen analysis was defined as “pathological” if at least one parameter was pathological, as “borderline” if at least one parameter was borderline (without any pathological parameters), and as “normal” if all three parameters were normal.

**Table 1:** Lower 5th percentile semen parameter values (WHO 2021 manual), terminology and “decision limits” definition

Sperm concentration (10^6^ per ml)	16		
Progressive motility (%)	30		
Normal morphology (%)	4		
Oligozoospermia	Low sperm concentration		
Athenozoospermia	Low progressive motility		
Teratozoospermia	Low normal morphology		
Normozoospermia	Normal semen parameters		
Oligoasthenoteratozoospermia	All semen parameters low		
Decision limits			
	Normal	Borderline	Pathological
Concentration (× 10^6^/mL)	≥ 20	10–20	< 10
Progressive motility (%)	≥ 50	35–49	< 35
Morphology (typical forms in %)	≥ 14	4–13	< 4

Basic characteristics recorded for both the male and the female partners were age, body mass index (BMI), and smoking status. Additionally collected data were duration and type of infertility (primary vs. secondary) as well as female serum hormone levels, including thyroid stimulating hormone (TSH), luteinizing hormone (LH), follicle-stimulating hormone (FSH), total testosterone, dehydroepiandrosterone-sulfate (DHEAS), sexual hormone binding globulin (SHBG) and Anti-Mullerian hormone (AMH). Serum blood samples were retrieved during the early follicular phase at days 2–5 of the menstrual cycle and analyzed according to ISO 15189 quality standards at the Department of Laboratory Medicine, Medical University of Vienna.

## Statistical analysis

Numerical variables are presented as median (interquartile range, IQR), categorical variables as number (frequency). For the comparison of categorical data, the Fisher’s exact test was applied. Correlations were tested using the Pearson correlation. Multivariable binary regression models were performed to evaluate factors associated with abnormal sperm test results. For these analyses, odds ratios (OR) with 95% confidence intervals (95% CI) as well as Nagelkerke’s R2 values for the goodness of fit are provided. For all analyses, the Statistical Package for the Social Sciences version 29.0 (IBM SPSS, Armonk, NY, USA) was used. *P* values of < 0.05 were considered statistically significant.

## Results

Basic characteristics of the females with PCOS or bilateral tubal occlusion and their male partners are provided in Table 2. Table 3 provides details about the results of semen analyses. In the PCOS group, 49/187 male partners (26.2%) had a completely normal result according to the decision limits, whereas 42 (22.5%) and 96 (51.3%) males revealed a borderline result or at least one pathological value, respectively. In the control group, 32/76 (42.1%) semen analyses results were classified as completely normal, 34 (44.7%) as borderline and 10/176 (13.2%) as pathological (*p* = 0.027).

**Table 2:** Basic patient characteristics of females and their male partners

		PCOS group (*n* = 187)	Control group (bilateral tubal occlusion; *n* = 76)	*p*
*Details about infertility*				
Duration of infertility (months)*		24 (12; 42)	21 (12; 36)	0.644
Primary infertility^b^		129 (69.0)	52 (68.4)	0.929
Women with polycystic ovarian morphology^b^		144 (77.0%)	0	< 0.001
Women with hyperandrogenism^b^		71 (38.0%)	0	< 0.001
Women with oligo-/anovulation^b^		187 (100.0)	0	< 0.001
*Male partners*				
Age (years)^a^		32 (28; 36)	35.5 (30.0; 39.0)	< 0.001
BMI (kg/m^2^)^a^		25.0 (23.8; 27.3)	24.7 (23.3; 27.4)	0.028
Weight category	Normal weight/underweight (BMI < 25.0 kg/m^2^)	93 (49.7)	38 (50.0)	0.807
	Overweight (BMI 25.0–30.0 kg/m^2^)	79 (42.2)	30 (39.5)	
	Obesity (BMI ≥ 30.0 kg/m^2^)^b^	15 (8.0)	8 (10.5)	
Smoker^b^		36 (19.3)	23 (30.3)	0.378
*Female partners*				
Age (years)^a^		32 (28; 34)	36 (30; 38)	0.005
BMI (kg/m^2^)^a^		26.7 (21.5; 30.1)	24.2 (21.7; 27.3)	0.600
Weight category	Normal weight/underweight (BMI < 25.0 kg/m^2^)	78 (41.7)	45 (59.2)	0.023
	Overweight (BMI 25.0–30.0 kg/m^2^)	63 (33.7)	21 (27.6)	
	Obesity (BMI ≥ 30.0 kg/m^2^)^b^	46 (24.6)	10 (13.2)	
Smoker^b^		22 (11.8)	12 (15.8)	0.052
LH^a^		10.5 (6.8; 17.0)	6.0 (4.2; 7.2)	< 0.001
FSH (mIU/mL)^a^		5.8 (4.7; 6.9)	6.4 (5.6; 7.2)	0.012
Testosterone (pg/mL)^a^		0.40 (0.30; 0.56)	0.25 (0.16; 0.31)	< 0.001
DHEAS (µg/mL)^a^		2.41 (1.79; 3.35)	2.19 (1.81; 2.80)	0.270
SHBG (nmol/L)^a^		38.5 (23.2; 66.7)	82.3 (68.0; 114.6)	< 0.001
AMH (ng/mL)^a^		7.4 (5.2; 11.3)	2.4 (1.7; 4.0)	< 0.001

Data are provided as ^a^ median (IQR) or ^b^ *n* (%)

**Table 3:** Semen analysis results

		PCOS group	Control group	*p*
Sperm concentration (× 10^6^/mL)^a^		49 (26; 82)	61 (38; 93)	0.127
Total sperm number (× 106/ejaculate)^a^		107 (48; 160)	96 (30; 154)	0.361
Progressive motility (%)^a^		53 (40; 64)	65 (43; 75)	0.001
Normal morphology (%)^a^		12 (8; 18)	9 (6; 11)	0.003
Concentration^b^	Normal	151 (80.7)	71 (93.4)	0.016
	Borderline	22 (11.8)	2 (2.6)	
	Pathological	14 (7.5)	3 (3.9)	
Progressive motility^b^	Normal	100 (53.5)	54 (71.1)	0.033
	Borderline	59 (31.6)	14 (18.4)	
	Pathological	28 (15.0)	8 (10.5)	
Morphology^b^	Normal	75 (40.1)	40 (52.6)	0.042
	Borderline	93 (49.7)	34 (44.7)	
	Pathological	19 (10.2)	2 (2.6)	
Overall result^b^	Normal	49 (26.2)	32 (42.1)	0.027
	Borderline	96 (51.3)	34 (44.7)	
	Pathological	42 (22.5)	10 (13.2)	

Data are provided as ^a^median (IQR) or ^b^*n* (%); ^c^multiple citations possible

*P* values are corrected for male age and male BMI

We then focused on factors associated with pathological sperm test results in the PCOS group (Table 4). When only male characteristics and the duration and type of infertility were entered into the model, higher male body mass index (BMI) and male smoking status were associated with a higher risk for an abnormal sperm test result (model 1, Nagelkerke’s R2 = 0.333). One could hypothesize that in one family, female and male BMI could be linked to each other based on shared dietary and lifestyle habits. Notably, female and male BMI were positively correlated (*r* = 0.532; *p* < 0.001 in Pearson correlation). However, assuming that in many cases one would have access to the female partner’s data, when only these variables were entered into model 2, the goodness of fit was lower (Nagelkerke’s *R*2 = 0.018), since neither the women’s age, nor BMI were significantly predictive (*p* > 0.05).

**Table 4:** Predictive factors for abnormal sperm test results in male partners of anovulatory PCOS women

	Semen analysis		Model 1		Model 2	
	Pathological (*n* = 42)	Normal or borderline (*n* = 145)	OR (95% CI)	p	OR (95% CI)	*p*
Male age (years)^a^	35 (30; 38)	32 (28; 35)	1.064 (0.979; 1.155)	0.144	–	–
Male BMI (kg/m^2^)^a^	27.4 (24.5; 29.4)	24.7 (23.8; 26.4)	1.478 (1.252; 1.745)	< 0.001	–	–
Male smoking^b^	17 (40.5)	19 (13.1)	6.228 (2.410; 16.098)	< 0.001	–	–
Female age (years)^a^	31 (29; 34)	32 (27; 35)	–	–	1.008 (0.932; 1.089)	0.847
Female BMI (kg/m^2^)^a^	28.9 (23.8; 30.6)	26.1 (21.3; 29.6)	–	–	1.054 (0.984; 1.130)	0.136
Duration of infertility (months)^a^	24 (12; 36)	24 (12; 48)	0.997 (0.982; 1.011)	0.656	1.001 (0.988; 1.1014)	0.880
Primary infertility^b^	29 (69.0)	100 (69.0)	0.864 (0.344; 2.173)	0.756	1.064 (0.494; 2.292)	0.875

Data are provided as ^a^median (IQR) or ^b^*n* (%)

## Discussion

Results of this study indicate that abnormal semen analysis results are quite common in male partners of women eligible for ovarian stimulation for anovulatory polycystic ovarian syndrome (PCOS). In this cohort, among male partners around one in five male partners (22.5%) demonstrated at least one pathological semen quality parameter, notably exceeding the 13.2% incidence observed in a control group of male partners of women with bilateral tubal occlusion. These results are of clinical relevance. Empirically, many men may underestimate the potential of male factor infertility when a severe reproductive pathology, such as anovulatory PCOS has been diagnosed in their female partner. Ovulatory dysfunction is a frequent etiology of female infertility, with PCOS accounting for up to 70–80% of anovulatory subfertility in women of reproductive age [2]. Given its high prevalence and adverse effects on fecundity, PCOS is among the most frequent endocrine disorders encountered by reproductive specialists [31]. The high prevalence of abnormal sperm analysis results found in this study cohort case–control highlights the importance of early male fertility evaluation, probably even before proceeding to PCOS-specific interventions such as ovarian stimulation.

At present, there exists one evidence-based guideline for the evaluation and treatment of polycystic ovary syndrome (PCOS) that has received endorsement by both the European Society of Human Reproduction and Embryology (ESHRE) and the American Society for Reproductive Medicine (ASRM). This recommendation is supported by numerous other esteemed societies and organizations specializing in endocrinology, reproductive medicine or gynecology and obstetrics across 71 countries [3]. From a global perspective, it can, therefore, be regarded as the primary source of reference for experts and physicians attending to patients with PCOS. In these guidelines, semen analysis is mentioned as a clinical recommendation only one time prior to tubal patency testing. Nevertheless, the routine inclusion of semen analysis in initial fertility evaluations seems to be prevailing consensus among reproductive specialists [10], and a further andrological evaluation of the male partner might be recommended if the results reveal abnormal sperm quality [6]. Scientific evidence to support this in women with anovulatory PCOS is insufficient, considering the lack of epidemiological data on the prevalence of impaired sperm quality in this population. Certainly, other guidelines do mention the importance of assessing male fertility before infertility treatments. For instance, the 2019 guideline for Diagnosis and Treatment Before Assisted Reproductive Treatments by the DGGG, OEGGG, and SGGG clearly recommends an andrological workup including semen analysis within 1 year of presentation for couples with an abnormal gynecological history. [32]. Similarly, the 2021 US-American AUA/ASRM guideline for male infertility advises simultaneous fertility evaluation for both partners before treatment initiation [11]. However, since these guidelines, along with others, are not specifically published for PCOS they may not be used as the primary reference when treating patients with this condition.

To provide a context for our results, male partners of women with bilateral tubal occlusion were selected as controls, due to a minimized the risk of selection bias related to pre-existing male infertility factors as compared to other couples seeking reproductive consultation. Moreover, semen analysis results of these couples may often be readily available due to their frequent visits to fertility clinics. This is an important factor, considering the absence of comprehensive data on the incidence of impaired or pathological semen quality in the general population. Most research investigating the epidemiology of suboptimal sperm quality merely provides mean values of semen parameters and/or changes within them over time. Further, nearly all prevalence studies on this topic are subject to selection bias by investigating only infertility clinic patients without further specifications or using semen donors. One of the very few studies providing numbers for potential extrapolation to the general population analyzed young, healthy military recruits from Baltic countries and found abnormalities in at least one semen quality parameter according to 2010 WHO reference values [23] with a prevalence of 11–15% of men [33]. However, the median age of this cohort was 19.8, with some men being as young as 16 years, thus not accurately mirroring the public. In an effort to minimize selection bias, another study conducted in 2009 aimed to examine the sperm quality in 304 couples with chronic anovulation in the female, and found abnormalities in 54% of male partner semen analysis results [34]. Like for much of the literature on this topic, however, the utilization of outdated reference values impairs the comparability of the results with more recent findings on male sperm quality. To the best of our knowledge, there are currently no other studies assessing the prevalence of abnormal semen quality in male partners of women with PCOS. However, Gao et al. assessed the predictive capacity of semen quality on conception, clinical pregnancy, and live birth rates in PCOS women with ovulatory dysfunction and provided data on basic sperm parameters of their male partners. However, this analysis included only men with sperm concentration and motility above certain reference values, impeding comparability to our results [35]. Given the increasing interest in establishing individual reference values and decision limits for semen analysis results with respect to particular reference populations [36], there is a growing need for more data on sperm quality parameters in specific patient cohorts, such as women with PCOS undergoing ovarian stimulation.

In general, epidemiologic studies on male infertility present significant challenges due to missing data regarding individual men without a partner or in same sex-relationships, as well as inconsistencies in defining, evaluating and distinguishing forms of infertility (e.g., primary versus secondary). Most importantly there is a significant dependence on female factors [37]. According to a meta-analysis, the global prevalence of male infertility is estimated to range between 2.5% and 12% [38]. It should be emphasized that the number of high-quality studies suitable for inclusion was limited.

While some studies suggest male infertility to be the most common diagnosis in couples failing to conceive, others demonstrated female factors to be predominantly causal. Such discrepancies may be attributed to varying definitions and assessment of male infertility and highlight the need for further research [37]. As cited in numerous publications, male factors are estimated to be responsible for 20–30% of infertility cases and contribute to some extent in up to 50% [6, 38]. This might seem high compared to 22% of abnormal semen analysis results found in our cohort. However, these statistics refer to a preselected cohort of infertile couples, where male factor infertility will be more prevalent without an apparent reproductive disorder in all female partners that could account for the infertility issues. Furthermore, there is a lack of robust data confirming these figures that originated from a study conducted in 1991 across three French regions, involving just 1686 couples seeking infertility treatment [39]. Considering the dated nature of much of the literature, it is challenging to draw reliable conclusions regarding the precise distribution of male and female factors responsible for infertility in couples [5, 38]. Although semen analysis can identify male subfertility only to some extent, there are currently no other clinically useful tests available that provide a comparably objective and standardized assessment of male reproductive function [40]. In general, semen quality can serve as an indirect marker for male fertility potential and eliminate certain limitations of direct epidemiologic studies. Basic semen parameters have shown good predictive potential for spontaneous conception [41] and live birth rates in couples using ovulation induction only or no treatment [42]. Moreover, the likelihood of male infertility correlates with a decrease in sperm cell concentration and the percentage of spermatozoa with normal morphology and motility [43, 44]. In 2010, the WHO released the fifth version of their laboratory manual for semen analysis, introducing clear lower reference limits for basic sperm parameters based on the 5th centile values of a fertile population [23]. These values, however, were abandoned in the most recent sixth WHO manual released in 2021 and replaced by “decision limits” that allow a classification of basic semen quality and quantity parameters into normal, borderline and pathological. This change should emphasize that applying threshold values in semen analysis is not meant to differentiate infertile from fertile semen, but to provide information for an initial evaluation of the male fertility status and guide further management. Moreover, reclassification of semen analysis results may broaden the eligibility for reproductive interventions, as many men whose results were previously classified as “normal” according to the 2010 WHO reference values will now fall into the “borderline” category [22, 25].

Considering the above-mentioned upper range of 12% for male infertility prevalence [38], 13.2% abnormal sperm quality in our control group, and lack of comparable studies, the rate of 22.5% semen analysis results with at least one pathological parameter found in our population of male partners of PCOS women seems considerably high. This appears highly relevant for counseling of PCOS patients before ovarian stimulation. Notably, differences in dietary habits between PCOS women and healthy female controls have been reported [13]. Noteworthy, 58.3% and 50.2% of women and men in our PCOS study population had a BMI > 25 kg/m^2^, respectively, placing them within the overweight range. A high rate of women with increased BMI might be seen as typical for a PCOS population, a clinical pattern where genetically determined metabolic factors as well as childhood and adolescent obesity are risk factors for its development [45]. However, the 50% rate of overweight males can be considered very high. It has been acknowledged that cultural practices, which promote social connection and include family life, are often characterized by low-nutrient, high-calorie foods [14, 15]. In addition to preferences in the choice of the partner, this could explain the significant positive correlation between the female and the male BMI values. Nonetheless, only basic characteristics of the male partners were predictive for the presence of at least one pathological sperm test results (higher age, higher BMI, smoking status; Table 4: model 1), neither age nor BMI of the woman. This is in line with previous findings on the negative association between male obesity [16], age and tobacco use [46] and sperm quality. It might be tempting to assess the male partner’s risk based on the female’s available data. However, this is not scientifically sound. Thus, to provide correct information about the risk for an abnormal semen analysis, one needs to know the basic characteristics of the male partner involved. Nonetheless, the predictive model 1 explains only a moderate proportion of abnormal findings (Nagelkerke’s R2 = 0.333). In conclusion, given a prevalence of more than 20% for a sperm test result with at least one pathological value, one might recommend a semen analysis before the first stimulation cycle to all couples with a PCOS woman.

The absence of information regarding reproductive outcomes might be addressed as a minor study limitation. Nevertheless, these outcomes would lack informative value, given that a considerable number of couples with significantly impaired semen quality opt for assisted reproductive techniques. Our findings are further limited by the presence of inconsistent definitions for male factor infertility and lack of consistent cut-off values in semen analysis. This continues to pose significant challenges for reproductive specialists when faced with the task of selecting the most suitable approach for fertility management in men with abnormal sperm quality. Importantly, evidence has shown considerable intra-individual variability in repeated semen analysis results in some subfertile couples [47]. For instance, a recent 2023 analysis by Boeri et al. revealed that around 60% of infertile men with an initial semen analysis above WHO limits exhibited results below these thresholds in a subsequent analysis [48]. Conversely, this study provides valuable information by highlighting such limitations of conventional semen analysis for the overall assessment of male fertility. Lastly, an apparent limitation is the retrospective study design and the moderate sample size. Examining a group of less than 200 males is considerably not big enough to deduce a sound prevalence. In contrast, the completeness of the data set with availability of all semen analysis parameters before the first PCOS-specific stimulation cycle, as well as for a control group, can be seen as strengths. In addition, we included a well-selected sample of a specific cohort of female PCOS patients, where corresponding literature is scarce.

## Conclusion

About 20% of male partners of infertile PCOS women eligible for initial ovarian stimulation with letrozole or clomiphene revealed an abnormal semen analysis result with at least one pathological value. This number was higher when compared to a control group of male partners of women with bilateral tubal occlusion. Our study offers new, valuable epidemiological information on the prevalence of impaired sperm quality in this population. Early semen analysis should, therefore, be self-evident in these patients to avoid unnecessary stimulation cycles and potentially indicate assisted reproductive technology (ART) at an earlier stage. Larger, probably prospective and multicentre studies with repeated semen analyses are needed to confirm our results and enable a definitive conclusion for clinical practice.

## Data Availability

Data are available upon reasonable request.
